# 作者更正

**Published:** 2021-09

由于本人疏忽，刊登于《中华血液学杂志》2021 年第1 期第21～26 页“普乐沙福联合G-CSF在浆细胞疾病自体造血干细胞动员中的有效性及安全性”一文中第22页右栏第5 行“普乐沙福：0.24 µg/kg”应为“普乐沙福：0.24 mg/kg”。特此更正。

作者：段文冰

